# Variation in vitamin D status in infants and children: a two-year cross-sectional study in Shanghai, China

**DOI:** 10.1186/s12887-023-04352-z

**Published:** 2023-10-26

**Authors:** Aiguo Li, Fang Wang, Ying Wu, Jiangfang Gao, Bosheng Li, Huiming Sheng, Jun Ma, Xiang-Peng Liao

**Affiliations:** 1 Department of Pediatrics, Tongren Hospital, Shanghai Jiao Tong University School of Medicine, Shanghai, China; 2 Department of Pediatrics, Shanghai Xuhui Central Hospital, Shanghai, China; 3 Department of Laboratory Medicine, Tongren Hospital, Shanghai Jiao Tong University School of Medicine, Shanghai, China; 4 Center for Community Health Care, China Hospital Development Institute, Shanghai Jiao Tong University, Shanghai, China

**Keywords:** Vitamin D, 25-hydroxyvitamin D, Deficiency, Insufficiency, Infants, Children, Shanghai

## Abstract

**Background:**

Vitamin D deficiency (VDD) is a public health problem. The variation in vitamin D status across regions and populations remains unclear, and there is a lack of consensus regarding the screening for VDD in individuals.

**Methods:**

Children who visited the hospital from January 2019 to December 2020 were included in this study. Serum 25-hydroxyvitamin D (25(OH)D) concentrations were measured using an enzyme-linked immunosorbent assay. The cutoffs for serum 25(OH)D concentrations to define deficiency, insufficiency, and sufficiency were < 20 ng/mL, 20–30 ng/mL, and ≥ 30 ng/mL, respectively.

**Results:**

A total of 7285 children aged 0–11 years were assessed; the mean 25(OH)D level was 31.4 ng/mL, and the median 25(OH)D level was 30.7 (interquartile range 24.4, 37.5) ng/mL. The 25(OH)D level declined with age in clinical visiting children aged 0–11 years, but maintained a consistently high level in health examination children aged 4–11 years. The percentages of 25(OH)D < 20 ng/mL and 25(OH)D < 30 ng/mL were 10.0% and 43.8%, respectively. Higher percentages of VDD were found in clinical visiting children than in health examination children within the 6–11-year group (53.3% vs. 14.7%) and winter (44.3% vs. 15.4%).

**Conclusion:**

Low vitamin D status (deficiency and insufficiency) was more common in clinic-visiting children than in health examinations, especially in schoolchildren and in the winter. The study implies the positive effects of vitamin D assessments included in child health checkups to optimize vitamin D status.

## Background

Vitamin D deficiency (VDD) is a global problem. Various factors associated with VDD have been reported differently. Age, sex, genetics and ethnicity, skin color, season, diet and nutrition, cultural background, lifestyle, and disease can affect vitamin D levels [1–4]. However, no organization recommends population-based screening for VDD. The Endocrine Society recommends screening for VDD in individuals at risk, but does not recommend population screening for VDD in individuals not at risk.

Meanwhile, different cutoffs for vitamin D status are currently adopted in different regions, and a range of accepted reference values is still lacking. To maximize the health effects of vitamin D, the Endocrine Society defines serum 25-hydroxyvitamin D (25(OH)D) concentrations for VDD, vitamin D insufficiency (VDI), and vitamin D sufficiency (VDS) with cutoffs of less than 20 ng/mL (50 nmol/L), 20–30 ng/mL (50–75 nmol/L), and more than 30 ng/mL (75 nmol/L), respectively. The American Academy of Pediatrics defines VDS as 25(OH)D > 20 ng/mL [5]. To ensure a large safety margin, the Canadian Paediatric Society [6] and the French expert consensus [7] suggest that the upper limit of 25(OH)D level recommended in general pediatric populations is more prudently below 60 ng/mL (< 150 nmol/L).

Scheduled health care is vital for children’s health. As the American Academy of Pediatrics recommended, the emphasis in China is especially on well-child visits during the first three years of life [8]; after six years of age, there are school-entry health examinations. Depending on children’s health status, and the availability and accessibility of health resources, there are no unified checkup items for children’s health examinations worldwide. While assessment of 25(OH)D levels is suggested in children with symptoms of rickets, systematic measurement of serum 25(OH)D concentration is currently not recommended in the general pediatric population [7, 9].

It is well known that vitamin D potentially affects many other cellular functions, such as cell growth, neuromuscular and immune function, and energy homeostasis [10–13]. The lack of consensus for screening recommendations to identify VDD in children is due to a critical gap in the evidence [14]. Meanwhile, to our knowledge, there have been no reports of vitamin D status in the general pediatric population in large samples in Shanghai, China. Thus, vitamin D assessment in our daily work is offered to parents as an option in health checkups and pediatric disease clinics. We supposed that children attending pediatric clinics might have lower vitamin D status than those in well-child checkups. Thus, this study aimed to provide an assessment and comparison of vitamin D status between the two different child populations, and to explore the contributing factors to the improvement of vitamin D status.

## Method

### Ethical approval

The study (hospital-based, cross-sectional observational survey) was conducted at Tongren Hospital affiliated with Shanghai Jiao Tong University School of Medicine in Shanghai. The study protocol was approved by the Institutional Review Board of Tongren Hospital affiliated with Shanghai Jiao Tong University School of Medicine (No. 201,913,501). Informed consent was obtained from parents or legal guardians in the study.

### Study population

Shanghai (31 degrees north latitude) is one of the largest cities in China, situated in south-eastern China. The Shanghai Tongren Hospital is a tertiary-care central hospital located southwest of downtown Shanghai. From January 2019 to December 2020, children aged less than 11 years who visited the hospital were included in this study. The children either visited the Department of Pediatrics for clinical services, which were almost due to certain infections, or the Department of Child Healthcare for health examinations, and they were classified as ‘Pediatric Department’ or ‘Child Healthcare Department’, respectively. We extracted the information from the decoded data set, including age, sex, visiting date, visiting type, and concentration of 25(OH)D.

### Sample collection and assessment of vitamin D status

The 25(OH)D assessment was included as one of the routine laboratory examination items of children’s health checkups offered at the guardians’ option. Depending on the guardian’s agreement, the 25(OH)D assessment was also offered to children visiting the pediatric clinics. Informed consent was obtained from all subjects involved in the study.

A small blood sample (40 µL) was collected from each participant using a finger stick. Serum samples were stored in a refrigerator at 4 °C and tested within three days. Using an enzyme-linked immunosorbent assay, serum 25(OH)D concentration was measured following the manufacturer’s instructions (IDS Ltd., Boldon Colliery, United Kingdom). The inter-assay and intra-assay coefficients of variation were < 10%, respectively. We also used mass spectrometry measurements for quality control. The cutoffs of VDD, VDI, and VDS are serum 25(OH)D levels (ng/mL) < 20, 20–30, and ≥ 30, respectively, according to the Endocrine Society’s clinical practice guidelines [2].

### Statistical analyses

The age of the participants was divided into four groups: infant (0–1 year), toddler (1–3 years), preschool (3–6 years), and school-age (6–11 years). Seasonality for sample collection was defined as four groups: spring (March, April, May), summer (June, July, August), autumn (September, October, November), and winter (December, January, February).

Analyses of the serum 25(OH)D concentrations revealed the presence of a skewed distribution, and descriptive statistics were determined: mean, minimum (min), maximum (max), percentile data (P25, P50, and P75), and percentages (%). The non-parametric Chi-square test was used to compare differences between groups. The association between sex, age, and seasonality with low vitamin D status was determined through multinomial logistic regression. Additionally, the figures were generated using the 25(OH)D concentration for age groups stratified by visiting type and seasonality.

The results were considered statistically significant when the 2-tailed p-value was below 0.05. The statistical analysis was performed using SPSS 26.0 (IBM Corp., Armonk, NY, the United States).

## Results

### Vitamin D levels by sex, age group, and seasonality between departments

We recruited 7285 young children aged 0–11 years for this study; of them, 1121 were from the Pediatric Department, and 6164 were from the Child Healthcare Department. For all participants, the mean 25(OH)D concentration was 31.4 ng/mL, and the median 25(OH)D concentration was 30.7 (interquartile range (IQR) 24.4, 37.5) ng/mL. The majority (94.3%) of the serum 25(OH)D measurements were within the range of 12–50 ng/mL. The prevalence of 25(OH)D levels beyond the range of 12–50 ng/mL differed significantly between departments (*p* < 0.001), with 4.4% and 2.2% of children from the Pediatric Department having 25(OH)D levels < 12 ng/mL and ≥ 50 ng/mL, respectively, compared to 1.2% and 4.4% in children from the Child Healthcare Department.

The serum 25(OH)D levels by sex, age group, and seasonality are detailed in Table 1. The serum 25(OH)D levels showed no significant difference by sex within either department or between the two departments. However, the levels differed significantly by age group within or between departments (all *p* < 0.001). For children from the Child Healthcare Department, the schoolchildren had the highest 25(OH)D level with a median of 32.9 (IQR 25.1, 41.6) ng/mL, while for children from the Pediatric Department, the infants had the highest 25(OH)D level, with a median of 33.2 (IQR 28.2, 39.9) ng/mL. Meanwhile, for children from both departments, the serum 25(OH)D levels in summer and autumn were significantly higher than those in spring and winter, respectively (all *p* < 0.001), and autumn had the highest level.

**Table 1:** Serum 25(OH)D levels (ng/mL) by sex, age group, and seasonality between departments

Variables	Pediatrics							Child Healthcare						
	N	Mean	Min	Max	P25	P50	P75	N	Mean	Min	Max	P25	P50	P75
All	1121	26.6	6.1	71.4	18.4	25.5	32.9	6164	32.3	4.3	94.2	25.6	31.3	38.1
Sex^a^														
Boys	663	26.5	6.1	60.2	18.5	25.5	32.8	3185	32.3	6.9	90.7	25.7	31.4	37.9
Girls	458	26.8	6.7	71.4	18.3	25.6	33.1	2979	32.3	4.3	94.2	25.4	31.3	38.4
Age (years)^b^														
0–1	190	34.0	6.3	56.7	28.2	33.2	39.9	1460	33.2	7.0	84.0	26.9	31.9	38.8
1–3	250	29.7	10.0	71.4	24.6	29.3	34.6	1973	30.3	4.3	83.4	24.2	29.4	34.9
3–6	315	24.1	6.7	58.9	17.7	23.0	28.8	2336	33.3	5.3	94.2	26.3	32.6	40.0
6–11	366	22.7	6.1	60.2	16.1	19.7	26.9	395	33.4	8.5	76.0	25.1	32.9	41.6
Seasonality^c^														
Spring	305	24.2	6.3	55.3	16.9	23.0	30.4	1599	31.3	7.0	94.2	24.9	30.3	36.9
Summer	403	27.5	6.1	61.0	19.8	26.0	33.7	1777	33.1	7.4	90.7	26.5	32.1	38.7
Autumn	239	29.6	9.7	71.4	20.8	28.2	35.0	1521	33.9	4.3	89.3	27.1	32.6	40.2
Winter	174	24.6	6.7	56.2	16.2	23.2	31.3	1267	30.5	5.2	84.0	23.5	30.0	36.4

^a^ The serum 25(OH)D levels had no significant difference by sex within the departments of Child Healthcare (*p* = 0.992) and Pediatrics (*p* = 0.957); but were significantly different between the departments (all *p* < 0.001);

^b^ The serum 25(OH)D levels were significantly different by age group within and between the departments (all *p* < 0.001);

^c^ The serum 25(OH)D levels were significantly different by seasonality within and between the departments (all *p* < 0.001)

**Fig. 1:**
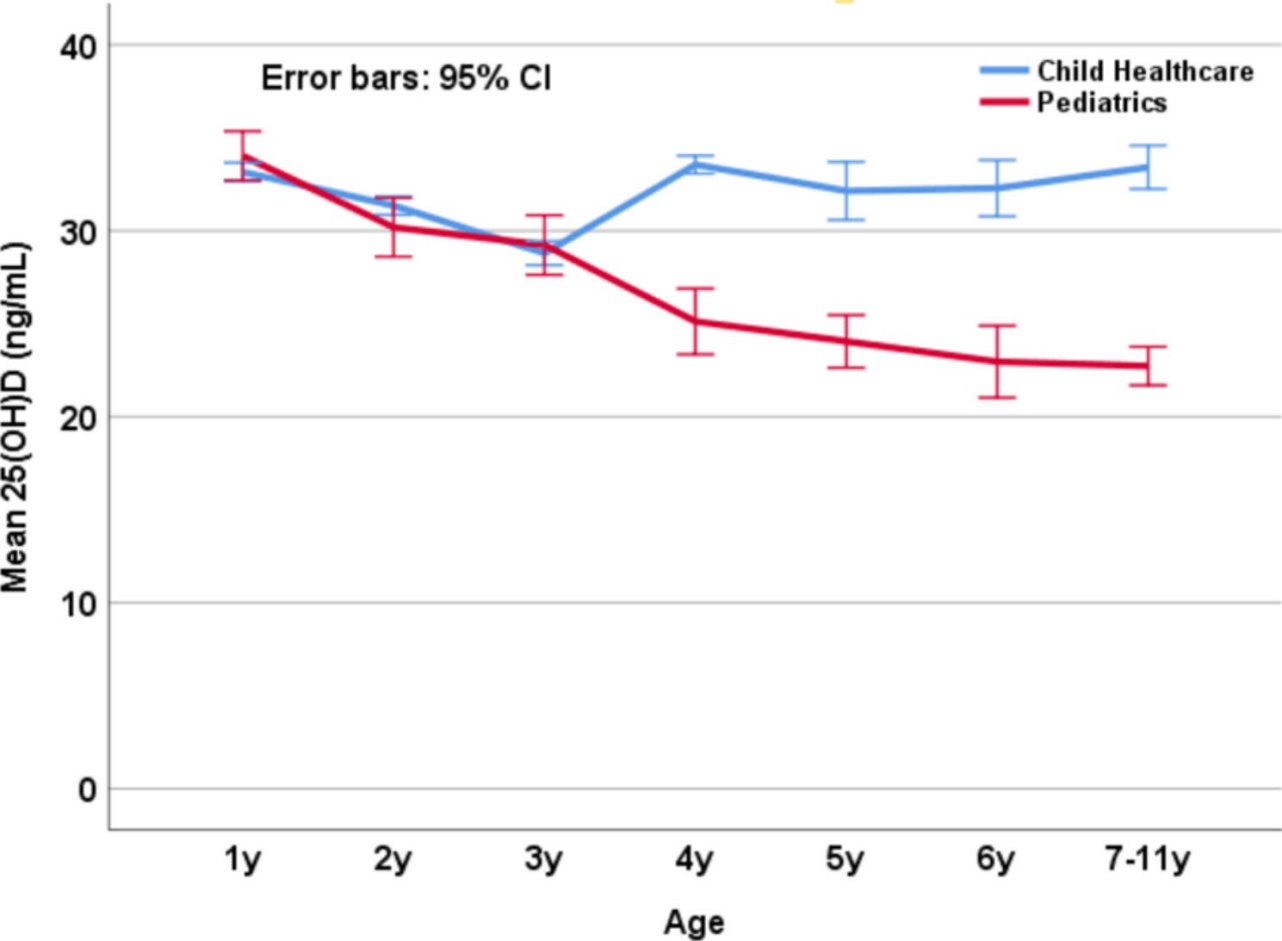
Serum 25(OH)D levels with age stratified by department of the sample source

**Fig. 2:**
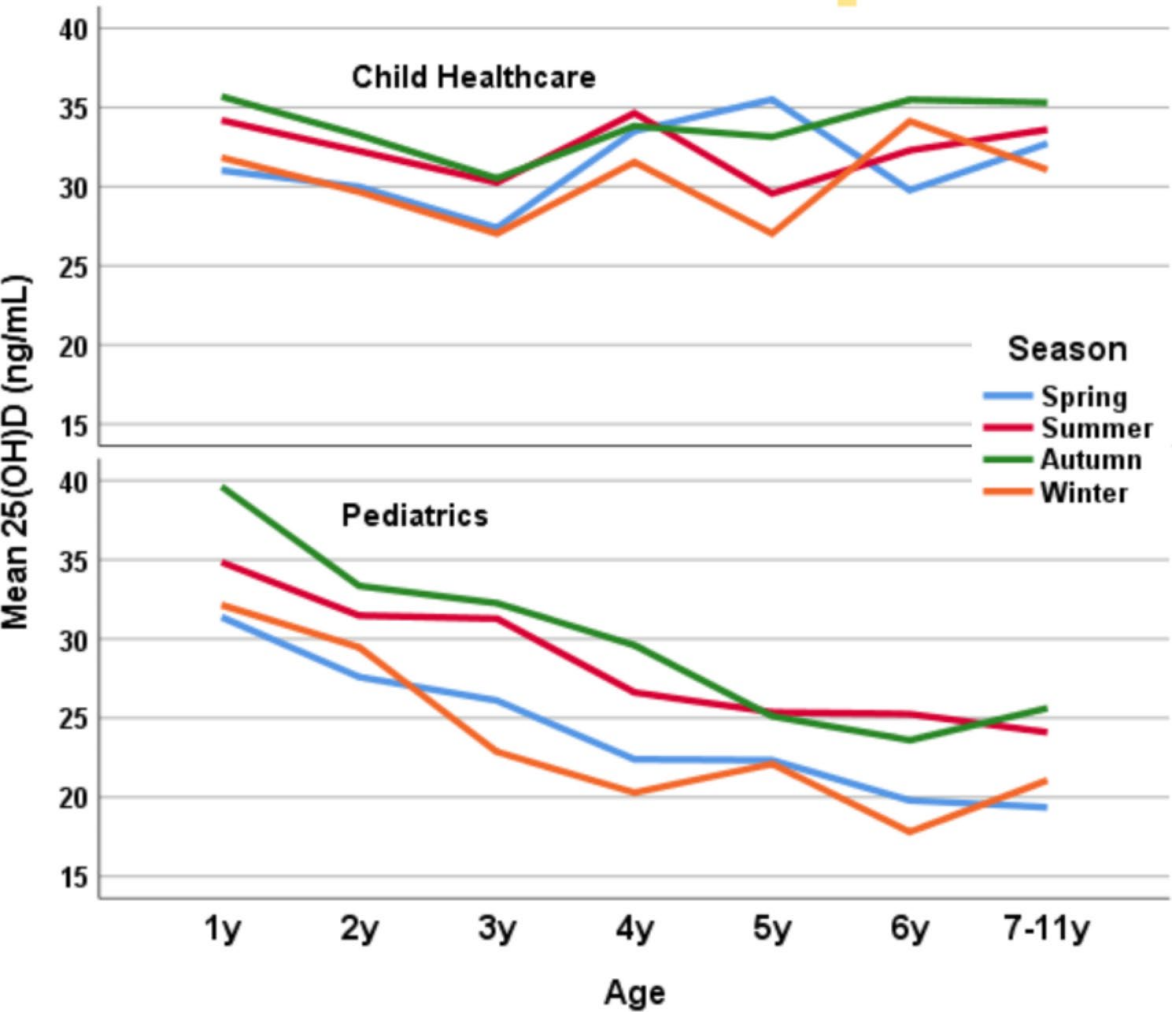
Serum 25(OH)D levels with age stratified by seasonality

Figure 1 illustrates the serum 25(OH)D tendency with age. There was a stable decrease in 25(OH)D levels in children from the Pediatric Department, but for children from the Child Healthcare Department, their 25(OH)D levels declined in 1–3 years, rose in 3–4 years, and maintained a steady high level in 4–11 years. Similarly, a decreasing tendency of 25(OH)D levels with age stratified by seasonality was shown in children from the Pediatric Department but not in those from the Child Healthcare Department (Fig. 2).

The 25(OH)D levels declined with age in the children from the Pediatric Department; however, for the children from the Child Healthcare Department, their 25(OH)D levels remained steady from 4 to 11years. The 25(OH)D levels between the two departments were not significantly different in the 1-year, 2-year, and 3-year groups, but were significantly different after three years of age (all *p* < 0.001).

For the children from the Child Healthcare Department and the Pediatric Department, serum 25(OH)D levels in summer and autumn were significantly higher than those in spring and winter, respectively (all *p* < 0.001). For the children from the Pediatric Department, a declining tendency of 25(OH)D levels with age was found in different seasons.

### Vitamin D status by sex, age group, and seasonality between departments

For all participants, the percentages of 25(OH)D < 20 ng/mL and 25(OH)D < 30 ng/mL were 13.4% and 47.3%, respectively; and the proportions of VDD, VDI, and VDS were 13.4%, 33.9%, and 52.7%, respectively. Vitamin D status was significantly different among age groups, seasons, and departments (all *p* < 0.001), but not between sexes. Higher percentages of VDD were found in children from the Pediatric Department than in the Child Healthcare Department in the 6–11 year group (53.3% vs. 14.7%) and winter (44.3% vs. 15.4%) (all *p* < 0.001) (Table 2).

**Table 2:** Vitamin D status in children between departments (*N* = 7285)^a^

Variables	Pediatrics				Child Healthcare			
	VDD	VDI	VDS	*P*	VDD	VDI	VDS	*P*
All	359 (32.0)	383 (34.2)	379 (33.8)		618 (10.0)	2083 (33.8)	3463 (56.2)	
Sex								
Boys	213 (32.1)	221 (33.3)	229 (34.5)	0.957	325 (10.2)	1053 (33.1)	1807 (56.7)	0.992
Girls	146 (31.9)	162 (35.4)	150 (32.8)		293 (9.8)	1030 (34.6)	1656 (55.6)	
Age (years)								
0–1	13 (6.8)	48 (25.3)	129 (67.9)	< 0.001	97 (6.6)	476 (32.6)	887 (60.8)	< 0.001
1–3	36 (14.4)	105 (42)	109 (43.6)		224 (11.4)	837 (42.4)	912 (46.2)	
3–6	115 (36.5)	133 (42.2)	67 (21.3)		239 (10.2)	680 (29.1)	1417 (60.7)	
6–11	195 (53.3)	97 (26.5)	74 (20.2)		58 (14.7)	90 (22.8)	247 (62.5)	
Seasonality								
Spring	120 (39.3)	612 (38.3)	81 (26.6)	< 0.001	173 (10.8)	612 (38.3)	814 (50.9)	< 0.001
Summer	105 (26.1)	576 (32.4)	147 (36.5)		124 (7.0)	576 (32.4)	1077 (60.6)	
Autumn	57 (23.8)	447 (29.4)	104 (43.5)		126 (8.3)	447 (29.4)	948 (62.3)	
Winter	77 (44.3)	448 (35.4)	47 (27.0)		195 (15.4)	448 (35.4)	624 (49.3)	

^a^ Values are expressed as n (%), and the data were analyzed with the Chi-square test. The cutoffs of VDD, VDI, and VDS are serum 25(OH)D levels (ng/mL) < 20, 20–30, and ≥ 30, respectively, according to the Endocrine Society guidelines

### Analysis of multinomial logistic regression

For predicting low vitamin D status among factors of sex, age, and seasonality between departments, the multinomial logistic regression model found that VDD was more common in winter and spring in both departments. Compared with autumn, higher risks of VDD in winter were found in children from both the Pediatric Department and Child Healthcare Department (odds ratio (OR) 4.95; 95% confidence interval (CI) 2.82–8.70 vs. OR 2.36; 95% CI 1.84–3.02) (Table 3).

**Table 3:** The association of sex, age, and seasonality with low vitamin D status in children between departments (N = 7285)^a^

Variables	Pediatrics				Child Healthcare			
	VDD (< 20 ng/mL)		VDI (20–30 ng/mL)		VDD (< 20 ng/mL)		VDI (20–30 ng/mL)	
	OR (95% CI)	*P*	OR (95% CI)	*P*	OR (95% CI)	*P*	OR (95% CI)	*P*
Sex								
Boys	REF							
Girls	1.35 (0.96, 1.90)	0.08	1.24 (0.92, 1.68)	0.16	0.99 (0.83, 1.18)	0.91	1.06 (0.95, 1.18)	0.32
Age (year)								
0–1	0.03 (0.01, 0.05)	< 0.001	0.24 (0.15, 0.39)	< 0.001	0.43 (0.30, 0.61)	< 0.001	1.43 (1.09, 1.86)	0.009
1–3	0.10 (0.06, 0.16)	< 0.001	0.68 (0.45, 1.03)	0.07	0.98 (0.71, 1.36)	0.91	2.46 (1.90, 3.19)	< 0.001
3–6	0.63 (0.41, 0.95)	0.03	1.53 (1.00, 2.34)	0.05	0.69 (0.50, 0.96)	0.03	1.30 (1.00, 1.68)	0.049
6–11	REF							
Seasonality								
Spring	4.22 (2.58, 6.90)	< 0.001	2.29 (1.48, 3.56)	< 0.001	1.62 (1.26, 2.07)	< 0.001	1.59 (1.36, 1.86)	< 0.001
Summer	1.20 (0.76, 1.87)	0.44	1.41 (0.96, 2.08)	0.08	0.84 (0.65, 1.10)	0.21	1.11 (0.96, 1.30)	0.17
Autumn	REF							
Winter	4.95 (2.82, 8.70)	< 0.001	1.90 (1.12, 3.20)	0.02	2.36 (1.84, 3.02)	< 0.001	1.47 (1.24, 1.73)	< 0.001

^a^ The multinomial logistic regression model includes all variables in this table, and the vitamin D status is classified according to the Endocrine Society

## Discussion

Our results showed that the median 25(OH)D concentration in the participants was 30.7 (IQR 24.4, 37.5) ng/mL, and 10.0% and 43.8% of children had a deficient and insufficient vitamin D status, respectively. These problems were more common in clinic-visiting children than in health examination children in the 6–11-year group (53.3% vs. 14.7%) and in winter (44.3% vs. 15.4%).

Our study suggests a much-improved vitamin D status for children in Shanghai. The prevalence of VDD has been reported worldwide [4]. Estimates of the prevalence of 25(OH)D < 20 ng/mL in the population from countries and regions were reported as 24% (United States) [15], 37% (Canada) [16], 40% (Europe) [17], and 30% (Africa) [18]. Compared with the results of age-specific vitamin D levels, our study also suggests a higher vitamin D status in the participants. For example, national studies from North America found that the percentages of 25(OH)D < 20 ng/mL were 7.1% in children aged 1–5 years, 13.7% in children aged 6–11 years in the United States [19], and 21.8% in Canadian children aged 9–13 years [16]. Studies from Southern European countries found that an estimated 22–52% of children and adolescents had 25(OH)D < 20 ng/mL [17, 20]. In Asia, a Japanese national study estimated that the prevalence of 25(OH)D < 20 ng/mL was 25.4% in children aged two years [21]. Studies also reported that the percentages of 25(OH)D < 20 ng/mL were 24% among preschoolers in Mexico [22], and 48% among children aged 2–5 years in New Zealand [23]. However, a higher vitamin D status was observed in five countries of Sub-Saharan Africa, where the prevalence of 25(OH)D < 20 ng/mL was 7.8% in children aged 0–8 years, with an overall median 25(OH)D of 31.0 (IQR 25.4, 37.7) ng/mL [24], to which, our result is roughly equal (median: 30.7 (IQR 24.4, 37.5) ng/mL).

Several factors related to urbanization may contribute to our study’s higher vitamin D status. It is known that skin color, season, diet and nutrition, cultural background, lifestyle, and disease may affect vitamin D status [1–4]. In Shanghai, a metropolis with a high population density, environmental pollution affects ultraviolet exposure; and high-rise buildings occupy a limited urban area, resulting in limited public outdoor activities [25]. Moreover, a study in Shanghai found that very few children had active lifestyles, and 25.5% of children and adolescents had sedentary behavior [26]. Thus, vitamin D synthesis in the human body could be adversely affected by these factors. Nevertheless, urban residents may have better socioeconomic status and health awareness; and urbanization can provide residents with better medical facilities and services and the convenience to access them. Studies also reported higher vitamin D levels for urban dwellers than for rural dwellers in Ireland and China [27, 28].

Other factors also contributed to the relatively higher vitamin D status in our study. Currently, vitamin D supplementation for children in Shanghai is a routine healthcare service, which the Chinese Medical Association recommends as similar to the American Academy of Pediatrics [5], and this practice is well implemented for local residents. Additionally, primary community hospitals here are responsible for guiding vitamin D supplementation for infants, toddlers, and preschoolers.

The study used a VDD cutoff of < 20 ng/mL, and the observed prevalence of VDD was 13.4% (10.0% in children from the Child Healthcare Department and 34.2% in children from the Pediatric Department). This prevalence appeared relatively low compared to studies in other countries. However, when a lower cutoff of < 12 ng/mL was applied, the prevalence dropped to just 1.2% in children from the Child Healthcare Department and 4.4% in children from the Pediatric Department. This lower prevalence may be attributed to the routine vitamin D supplementation provided in healthcare practices. In addition, as Shanghai is a metropolis with a diverse population, some children are not used to vitamin D supplementation. Meanwhile, some lifestyle and disease factors may lead to low vitamin D levels.

The variation in vitamin D status among the children is worth mentioning. As illustrated in Fig. 1, we found that the serum 25(OH)D levels between the two departments were roughly similar in the participants less than three years old, but different after three years old. This variation may be due to several factors: first, the children after three years old took their healthcare examination less frequently, and their parents’ awareness of vitamin D supplementation for children could be slackened. Also, the variation may be associated with the progress of vitamin D guidelines in China, where vitamin D supplementation was previously stressed for children less than three years old [29, 30]. Furthermore, the percentage of children attending health checkups is an assessment indicator for managing children aged 0–3 years by the children’s health authority in Shanghai, China. Thus, an overall decline in vitamin D levels is observed, especially in children visiting pediatric clinics (Table 1; Fig. 1, and Fig. 2). However, for the children in the healthcare group, a steady optimal vitamin D status with age was found, and the difference in this comparison suggests the effects of scheduled healthcare checkups with 25(OH)D assessment on sustaining optimal vitamin D status in children.

Our study also offers more data on the value of serum 25(OH)D assessment in different pediatric populations. It is generally acknowledged that population-based screening with serum 25(OH)D is not indicated [9], and measurement of serum 25(OH)D levels is recommended when there are symptoms of rickets [7, 14]. In practice, it is generally the case that if vitamin D is routinely supplemented in all infants and children, routine vitamin D assessment may not be necessary. It might be more efficient to focus on monitoring vitamin D levels in specific cases, such as children with illnesses or those whose compliance with supplementation is questionable. In our practice, assessing 25(OH)D levels in children is both affordable and acceptable, with a cost of less than ten dollars for each test. Meanwhile, the report showed that the overall prevalence of serum 25(OH)D < 50 ng/mL ranged from 24 to 49%, and global differences in vitamin D intakes depended on the world region [31]. Moreover, there is insufficient evidence for healthcare providers to recommend who should be screened for VDD [14]; thus, our study can provide more evidence for serum 25(OH)D assessment included in different pediatric populations. Further studies are warranted to develop vitamin D screening tools based on risk factors for children to maximize the impact of vitamin D on health [3, 14].

The strengths of this study include that it represents a large sample size with health examination children and clinical visiting children covering the age range of 0–11 years. However, there were several limitations. One of the limitations was the lack of anthropometric measurements, blood levels of calcium, phosphorus and parathyroid hormone, sociodemographic characteristics, sunlight exposure, diet, and vitamin D supplementation, which affect serum 25(OH)D concentrations. For some of the children from the Pediatric Department, only weight measurements were available, and height/length and blood levels of calcium and phosphorus were missing for the children. We also encountered barriers in exporting measurements from the electronic medical record system. As a result, we were unable to analyze the anthropometric measurements and disease factors associated with vitamin D status, which are the subject of further study. Additionally, we used the diagnostic criteria according to the Endocrine Society’s clinical practice, and different results may be obtained if different guidelines are used. Meanwhile, regular healthcare checkups are required by the child health policy in China. To our knowledge, this is the most extensive sample-size study of vitamin D status in children in Shanghai. Given this hospital-based survey with a sample size of 7285 children, including 6164 children for routine health checkups, the participants were representative of the children in Shanghai.

## Conclusion

This study identifies a high prevalence of low vitamin D status in Shanghai children aged 0–11 years, especially among schoolchildren and in the winter. Nevertheless, compared to literature reports, the vitamin D status of children in Shanghai is relatively higher. Moreover, vitamin D assessments included in child health checkups can assist in optimizing vitamin D status in children.

## Data Availability

The datasets supporting the conclusions of this article are not publicly available but are available from the corresponding author on reasonable request.

## Abbreviations

25(OH)D: 25-hydroxyvitamin D
VDD: Vitamin D deficiency
VDI: Vitamin D insufficiency
VDS: Vitamin D sufficiency
IQR: Interquartile range
CI: Confidence interval
OR: Odds ratio
